# Supplementary material for: Assessing single-locus CRISPR/Cas9-based gene drive variants in the mosquito Aedes aegypti via single-generation crosses and modeling
Source: G3 (Bethesda). 2022 Oct 17;12(12):jkac280. doi: 10.1093/g3journal/jkac280 (PMC9713460; doi:10.1093/g3journal/jkac280)
Supplement: jkac280_Supplementary_Data [file jkac280_supplementary_data.pdf]

Supplementary Information for:

Assessing single-locus CRISPR/Cas9-based gene drive variants in the mosquito *Aedes aegypti* via single generation crosses and modeling

William Reid, Adeline E Williams, Irma Sanchez-Vargas, Jingyi Lin, Rucsanda Juncu, Ken E Olson, Alexander WE Franz*

*Corresponding author: Alexander WE Franz
Email: franza@missouri.edu

This file includes:

Figure S1. Crossing schematic to test for maternal contributions of CRISPR/Cas9 components in the AeaNosC109^{GD} and AeaZpgC109^{GD} lines.
Figure S2. Size distributions of nucleotide insertions/deletions associated with NHEJ events observed in GD lines AeaNosT4^{GD}, AeaNosC109^{GD}, and AeaZpgC109^{GD}.
Figure S3. Modeling simulations for the AeaNosC109^{GD} line (unmodified upper row) allowing for changed parameters for pupation success (xiF and xiM, second row), female deposition rate (dF, third row), and GDBI development rates (crF and crM, third row).

Table S1. NCBI accession numbers for the constructs used in this study.
Table S2. List of primers and gBlocks used in this study.
Table S3. Life parameter data for hemizygote AeaNosC109^{GD} and AeaZpgC109^{GD} to assess fitness costs.
Table S4. Script modifications to the MGDrivE v1.6.0 Cube-CRISPR2MF.R to condense the B and R resistance alleles to a common no-fitness cost R allele.
Table S5. Fitness, GD, maternal deposition, and GDBI formation parameters used in the MGDrivE modeling for the AeaNosC109^{GD} and AeaZpgC109^{GD} lines.
Table S6. Polymorphisms in the active target sites for the Carb109 and TIMP-P4 loci among 132 genomes of *Aedes aegypti*.
Table S7. Numbers of larval pools assessed and average metrics for GD inheritance for the OX-1 crosses.
Table S8. Numbers of larval pools assessed and average metrics for GD inheritance for the OX-2 crosses.
Table S9. Percentage of amplicons containing gene drive blocking indels (GDBI) for pooled negative larvae from the OX-2 generations.

SI References

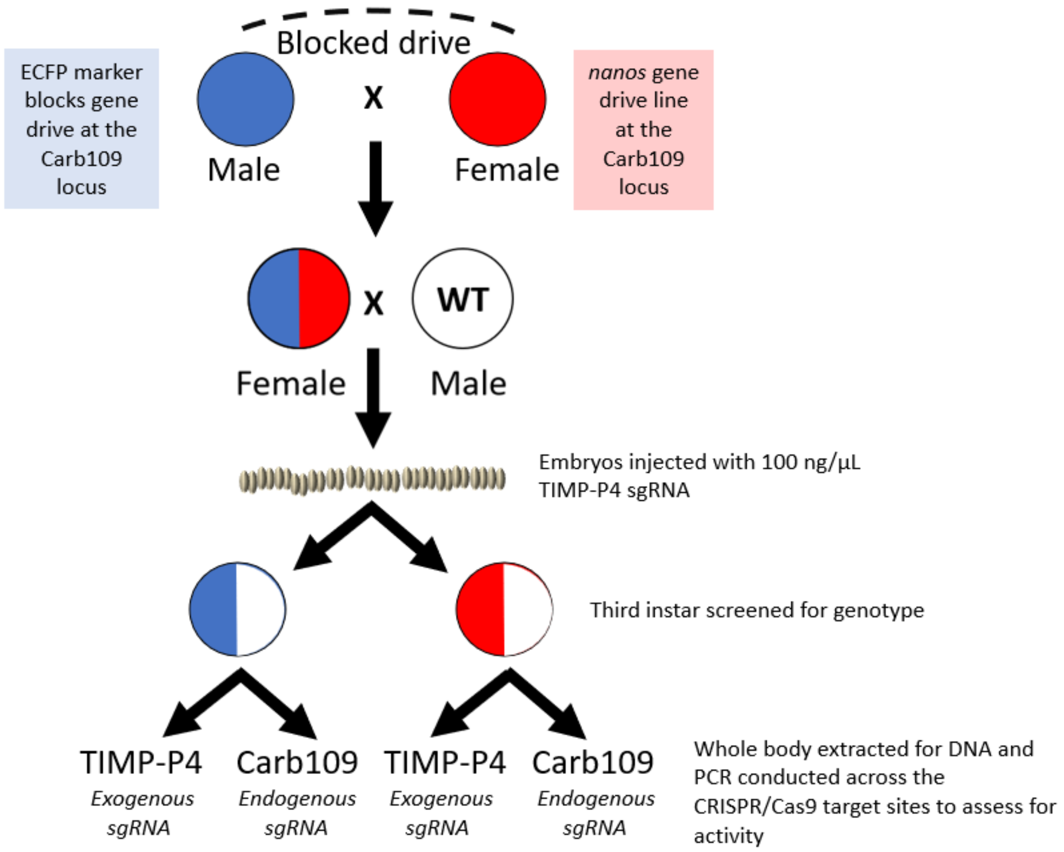

Figure S1. Crossing schematic to test for maternal contributions of CRISPR/Cas9 components in the AeaNosC109^{GD} and AeaZpgC109^{GD} lines. GD lines (mCherry marked) were balanced against a blocked GD (eCFP present at the CRISPR/Cas9 target site), and female trans-heterozygotes were then outcrossed to non-transgenic males. The embryos from this cross were subsequently injected with sgRNA targeting the TIMP-P4 locus, and the surviving larvae were reared to third instar, genotyped, and assayed for CRISPR/Cas9 activity at both the Carb109 locus (GD target) and the TIMP-P4 locus (exogenously applied sgRNA) using PCR and Sanger sequence trace analysis.

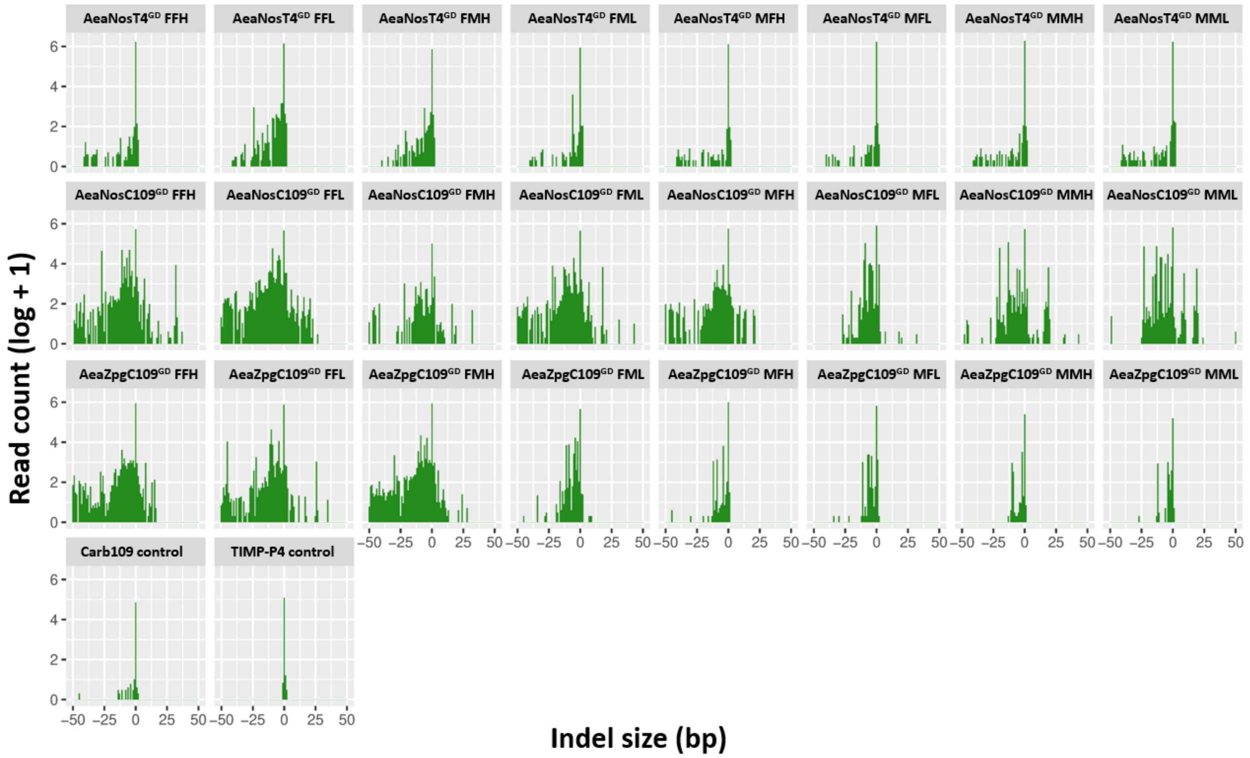

Figure S2. **Size distributions of nucleotide insertions/deletions associated with NHEJ events observed in GD lines AeaNosT4^{GD}, AeaNosC109^{GD}, and AeaZpgC109^{GD}.** The first letter within each cross indicates the parental transgenic sex, the second letter indicates the grandparental transgenic sex. L = low drive level in the parental generation, H = high drive in the parental generation. Control = Higgs' white eye (HWE) non-transgenic mosquitoes. Data are presented as a log transformation.

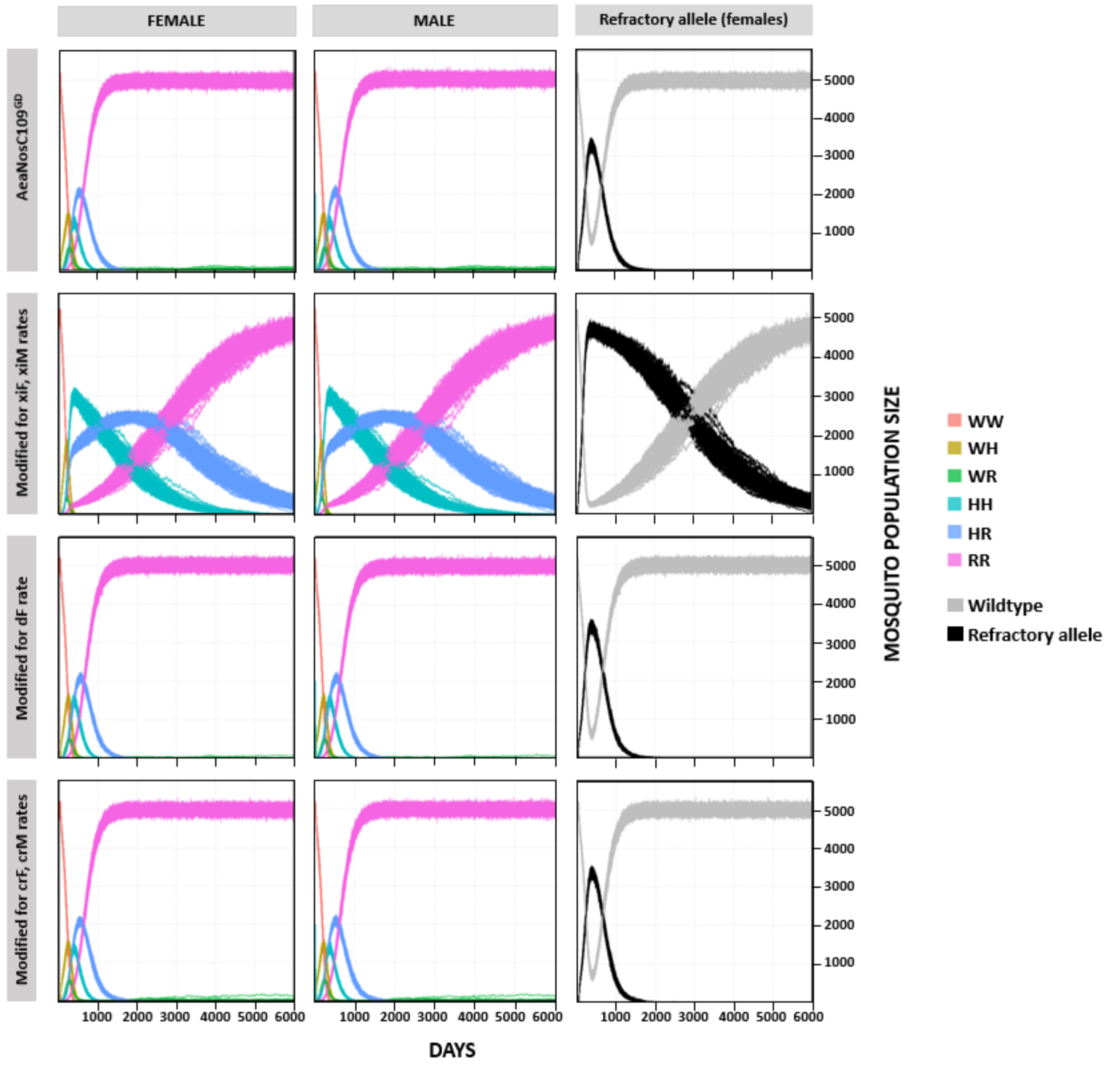

Figure S3. Modeling simulations for the AeaNosC109^{GD} line (unmodified upper row) using MGDrivE allowing for changed parameters regarding pupation success (xiF and xiM, second row), female deposition rate (dF, third row), and GDBI development rates (crF and crM, third row).

Table S1. **NCBI accession numbers for the constructs used in this study.**

Construct	Purpose	NCBI accession
AeaeCFPT4	Test knock-in plasmid for the TIMP-P4 locus	MT926371
AeaeCFPC109	Test knock-in plasmid for the Carb109 locus	OL452018
AeaNosT4^{GD}	Gene drive line for nanos Cas9 in the TIMP-P4 locus	OL452014
Aeaβ2tC109^{GD}	Gene drive line for β2tubulin Cas9 in the Carb109 locus	OL452017
AeaNosC109^{GD}	Gene drive line for nanos Cas9 in the Carb109 locus	OL452015
AeaZpgC109^{GD}	Gene drive line for zpg Cas9 in the Carb109 locus	OL452016
pAeU6-MT	AAEL017774 promoter with inverted *Bbs*I sites and chiRNA (Dang *et al*., 2015) (1)	OL452019
pAeT7ku70dsRNA	Template plasmid for the anti-ku70 dsRNA trigger from Basu *et al*. (2015) (2) flanked by T7 RNA promoters.	OL452021

Table S2. List of primers and gBlocks used in this study.

Primer	Name	Sequence (5’ -> 3’)
BR-26	AAEL017774PROMF	CCATCTAGAGACTGGTCTTTTAGTTATGACTTCTG
BR-27	AAEL017774PROMR	TTCGGATCCGAAGACCCATTTCACTACTCTTGCCTCTGC
BR-28	AAEL019894_3pUTRF-SalI	CATGTCGACAAGCCTAGAAGGAATAAAAATGGCTGAAATTATCCG
BR-29	AAEL019894_3pUTRR-XbaI	GCATCTAGAATCACTCTTACGACACTGCATAC
BR-32	AAEL019894PF_HindIII	CATAAGCTTAAACTCGAGTCGTGTGGCAAGTACGAAG
BR-33	AAEL019894PR_PstI-NcoI	CATCTGCAGCCATGGTGGAGCACTTCTAGCGGTTC
BR-34	AG45945F-NcoI	CATCCATGGACTATAAGGACCACGACG
BR-34	AG45945F-NcoI	CATCCATGGACTATAAGGACCACGACG
BR-35	AG45945R-SalI	CATGTCGACTTACTTTTTCTTTTTTGCCTGGCC
BR-35	AG45945R-SalI	CATGTCGACTTACTTTTTCTTTTTTGCCTGGCC
BR-40	ZA_AeNosPR-XhoIF	CATCTCGAGCACTATCAAACCCCTAAAGACA
BR-41	ZA_AeNosPR-PciIR	CATCTCGAGACATGTTGTTCGTTGATCTCGATCAGCCA
BR-42	ZA_AeNos3pU-StuIF	CATAGGCCTCGTAATCGAAGTGTTGGACGGGGAAAG
BR-43	ZA_AeNos3pU-NheIR	CATGCTAGCACCAGACATCCGTTTAAGCTGAACCAC
BR-44	ku70-ZA2341T7-F	TAATACGACTCACTATAGGGTTTCGCTGGGTGTCAACATTTATTCC
BR-45	ku70-ZA2342T7-R	TAATACGACTCACTATAGGGCTTGATTTTCTTCGCTACTGACTTGA
BR-54	3xP3FOR_XhoIF	CATCTCGAGAAATTCGAGCTCGCCCGG
BR-55	SV40REV_XhoIR	CATCTCGAGCCGTACGCGTATTCGATAAG
BR-60	Carb109scrF_3161	CGCACCTAATCAGACAGTCG
BR-80	T7_dsRNA_HindIII	CATAAGCTTAATACGACTCACTATAGGG
BR-98	SV40_GibREV	AGCGGCCGCGACTCTAGA
BR-99	3xP3_GibFOR	GGTGGCGACCGGTGGATC
BR-100	TIMP4donBK-R	GATCTAGAGTCGCGGCCGCTTTACTTGTACAGCTCGTCCATG
BR-101	TIMP4donBK-F	ACGATCCACCGGTCGCCACCATGGTGAGCAAGGGCGAG
BR-102	Blac-GibFOR	CTGCTATGTGGCGCGGTATTATC
BR-103	Blac_GibREV	GATAATACCGCGCCACATAGCAG
BR-112	Carb109_g15LAHR	CAATCTCGAGCCAGGGTCAGGGAGTTGGA
BR-113	Carb109_g15RAHF	CAATCTCGAGCTTTTCTTCGGCATATCTATCGCTAGG
BR-349	U6empty_Gblock	TCCACATCTCCATACATTCAACGCACTGTGCGGCTGTGCTGTGCGACTCCGTCGAGTCGACCAACATAGTTGAAACAAATTGAATATTTAATTGATCGTTATAGGAATGGTGTTAGATGAGTCATCCTTTACAGTAAGCACATACAGTATTATAATTGAAGATCGTCGGCAGATAGGTGTGTAGGGTAGAGTATCAGCAATAAGTTGGGACGTTTGACTTTTTGTAGGTAGACAAAAACTAAACTTTTTTTCGCTTCTCTATGTGTGCCCCCCCGGGTAGCGTATCGTTCCGATTGTGGTGCGAACGAATGAAATCGCCTATCGAGTTGATACGTCCATCTATCGCTAGAACCGCGTTCGCTGTAAAAGACTATATAAGAGCAGAGGCAAGAGTAGTGAAATGGGTCTTCGAGAAGACCTGTTTCAGAGCTATGCTGGAAACAGCATAGCAAGTTGA

		AATAAGGCTAGTCCGTTATCAACTTGAAAAAGTGGCACCGAGTCGGTGCTTTTTTTTTTTT
BR-350	SacII-U6MT-F	CAATATCCGCGGTCCACATCTCCATACATTCAACG
BR-351	SacII-U6MT-R	TAGATGCCGCGGAAAAAAAAAAAAAAAAGCACCGACTCGGTGCCAC
BR-360	TIMP4-Pstop	AAATGGACCAACCGCAGTCATTGTG
BR-361	TIMP4-Psbot	AAACCACAATGACTGCGGTTGGTCC
BR-362	C109-Pstop	AAATGGATATGCCGAAGAAAAGCCA
BR-363	C109-Psbot	AAACTGGCTTTTCTTCGGCATATCC
BR-364	C109RAH-SacII-R	CAATACCGCGGATGGGATGCAGAACCATTTG
BR-368	KpnI-Carb109scrF_3161	CAATAGGTACCGCACCTAATCAGACAGTCG
BR-655	zpgPROM-R_BsbI-PstI	CATTATCTGCAGGAAGACCCGATGATTTAGGGGTTTG
BR-656	zpgF_131-XhoI	CAATACTCGAGATGAATCCTAAAGTCCTGCTCG
BR-660	zpg3PUTR-F_BbsI-PstI	AAGTAACTGCAGGAAGACCTTCGATAAAAGTATCGTCCTAAGACTTATTCAG
BR-666	Cas9F-GG-ZPGredo	CAATATGAAGACGGCATCATGGACTATAAGGACCACGACG
BR-667	Cas9R-GG-ZPGredo	CAATATGAAGACGGTCGATTACTTTTTCTTTTTTGCCTGGCCG
BR-683	inx4-GIB_Rev	TGGACATGCATTAGACTTACTAGTAGGTGTTTGGACCAAGTGGAG
BR-724	C109NGS-F	ACACTCTTTCCCTACACGACGCTCTTCCGATCTCGCACCTAATCAGACAGTCG
BR-725	C109NGS-R	GTGACTGGAGTTCAGACGTGTGCTCTTCCGATCTCCTGCCTTCATTAAGCTCTTTG
BR-726	TIMPNGS-F	ACACTCTTTCCCTACACGACGCTCTTCCGATCTAACGAGATGCCTTCTCCTGA
BR-727	TIMPNGS-R	GTGACTGGAGTTCAGACGTGTGCTCTTCCGATCTAAAATGGCGTTCGATGAGA

Table S3. Life parameter data for hemizygote AeaNosC109^{GD} and AeaZpgC109^{GD} to assess fitness costs. Data derived from hemizygous males or HWE males that were allowed to mate with female HWE. * = 0.01 < *p* < 0.05 compared to HWE by one-tailed t-test. No stars indicate no significant difference by one-way ANOVA.

Line	WT (HWE)	AeaNosC109^{GD}	AeaZpgC109^{GD}
larva-to-pupa development (in days)	9.8 ± 4.3	9.5 ± 5.6	9.5 ± 7.0
larva viability (% survival)	44.2 ± 7.3	30.9 + 2.9*	44.8 ± 2.5
% female adults	45.4 ± 1.6	47.8 ± 1.5	48.2 ± 1.1
% male adults	54.6 ± 1.7	52.2 ± 1.5	51.8 ± 1.1
50% female survival (days)	34 ± 6.2	44.5	41.5
50% male survival (days)	20.7 ± 3.3	16 ± 1.6	18 ± 4.5
fecundity (# eggs)	62.6 ± 2.8	57.6 ± 2.6	64.6 ± 2.0
fertility (egg hatchability) (%)	49.2 ± 8.2	44.0 ± 3.6	58.3 ± 3.4
male contribution (# pos / total n)		35.5 ± 1.2 (1931/5447)	25.6 (1035/4049)

Table S4. Script modifications to the MGDrivE v1.6.0 Cube-CRISPR2MF.R to condense the B and R resistance alleles to a common no-fitness cost R allele. Original section of script taken from Sánchez *et al.* (2020) (3).

```
##REMOVED B GENOTYPES IN THE GTYPE VECTOR
 ## define matrices
 ## Matrix Dimensions Key: [femaleGenotype,maleGenotype,offspringGenotype]
 gtype <- c('WW', 'WH', 'WR', 'HH', 'HR', 'RR')
 size <- length(gtype)
 tMatrix <- array(data=0, dim=c(size, size, size), dimnames=list(gtype, gtype, gtype))
#transition matrix

 ## fill tMatrix with probabilities
 ## COMMENTED OUT ANY CROSSES WITH A B ALLELE AND THEN REDID ANYTHING IN
THE PROBABILITIES THAT WOULD GIVE A B ALLELE
 #('WW', 'WH', 'WR', 'HH', 'HR', 'RR')
 tMatrix['WW','WW', 'WW'] <- 1

 tMatrix['WR','WW', c('WW', 'WR')] <- c( 1, 1)/2
 tMatrix['WR','WR', c('WW', 'WR', 'RR')] <- c( 1/2, 1, 1/2)/2

 tMatrix['HH','HH', 'HH'] <- 1

 tMatrix['HR','HH', c('HH', 'HR')] <- c( 1, 1)/2
 tMatrix['HR','HR', c('HH', 'HR', 'RR')] <- c( 1/2, 1, 1/2)/2

 tMatrix['RR','WW', 'WR'] <- 1
 tMatrix['RR','WR', c('WR', 'RR')] <- c( 1, 1)/2
 tMatrix['RR','HH', 'HR'] <- 1
 tMatrix['RR','HR', c('HR', 'RR')] <- c( 1, 1)/2
 tMatrix['RR','RR', 'RR'] <- 1

 ## set the other half of the matrix that is symmetric
 # Boolean matrix for subsetting, used several times
 boolMat <- upper.tri(x = tMatrix[ , ,1], diag = FALSE)
 # loop over depth, set upper triangle
 for(z in 1:size){tMatrix[ , ,z][boolMat] <- t(tMatrix[ , ,z])[boolMat]}


 ## fill asymmetric parts of tMatrix
 #female specific homing, except for WHxWH

 tMatrix['WH','WW',] <- c((1-cF)*(1-dF), (1+cF*chF)*(1-dF), ((1-cF)*dF*drF + (cF*(1-
chF)*crF)*(1-dF)) +((1-cF)*dF*(1-drF) + (cF*(1-chF)*(1-crF))*(1-dF)),
```

```
(1+cF*chF)*dF*dhF, ((1+cF*chF)*dF*(1-dhF)*drF) + ((1+cF*chF)*dF*(1-dhF)*(1-drF)),
((cF*(1-chF)*crF)*dF*drF)+((cF*(1-chF)*crF)*dF*(1-drF) + (cF*(1-chF)*(1-
crF))*dF*drF)+((cF*(1-chF)*(1-crF))*dF*(1-drF)))/2

 tMatrix['WH','WH',] <- c((1-cF)*(1-cM)*(1-dF),
                         (1+cF*chF)*(1-cM)*(1-dF) + (1-cF)*(1+cM*chM),
                         ((1-cF)*(1-cM)*dF*drF + cF*(1-chF)*crF*(1-cM)*(1-dF) + (1-cF)*cM*(1-
chM)*crM)+((1-cF)*(1-cM)*dF*(1-drF) + cF*(1-chF)*(1-crF)*(1-cM)*(1-dF) + (1-
cF)*cM*(1-chM)*(1-crM)),
                         (1+cF*chF)*(1-cM)*dF*dhF + (1+cF*chF)*(1+cM*chM),
                         ((1+cF*chF)*(1-cM)*dF*(1-dhF)*drF + cF*(1-chF)*crF*(1+cM*chM) +
(1+cF*chF)*cM*(1-chM)*crM)+((1+cF*chF)*(1-cM)*dF*(1-dhF)*(1-drF) + cF*(1-
chF)*(1-crF)*(1+cM*chM) + (1+cF*chF)*cM*(1-chM)*(1-crM)),
                         (cF*(1-chF)*crF*(1-cM)*dF*drF + cF*(1-chF)*crF*cM*(1-
chM)*crM)+(cF*(1-chF)*crF*(1-cM)*dF*(1-drF) + cF*(1-chF)*(1-crF)*(1-cM)*dF*drF +
cF*(1-chF)*(1-crF)*cM*(1-chM)*crM + cF*(1-chF)*crF*cM*(1-chM)*(1-crM))+(cF*(1-
chF)*(1-crF)*(1-cM)*dF*(1-drF) + cF*(1-chF)*(1-crF)*cM*(1-chM)*(1-crM)))/4

 tMatrix['WH','WR', ] <- c((1-cF)*(1-dF), (1+cF*chF)*(1-dF), ((1-cF)*dF*drF + (cF*(1-
chF)*crF)*(1-dF) + 1-cF) + ((1-cF)*dF*(1-drF) + (cF*(1-chF)*(1-crF))*(1-dF)),
(1+cF*chF)*dF*dhF, ((1+cF*chF)*dF*(1-dhF)*drF + 1+cF*chF ) + ((1+cF*chF)*dF*(1-
dhF)*(1-drF)), ((cF*(1-chF)*crF)*dF*drF + cF*(1-chF)*crF) + ((cF*(1-chF)*crF)*dF*(1-
drF) + (cF*(1-chF)*(1-crF))*dF*drF + cF*(1-chF)*(1-crF)) + ((cF*(1-chF)*(1-crF))*dF*(1-
drF)))/4

  tMatrix['WH','RR',c('WR', 'HR', 'RR')] <- c(1-cF, 1+cF*chF, (cF*(1-chF)*crF)+(cF*(1-
chF)*(1-crF)))/2

 tMatrix['WH','HH',c('WH', 'HH', 'HR')] <- c(1-cF, 1+cF*chF, (cF*(1-chF)*crF)+(cF*(1-
chF)*(1-crF)))/2

 tMatrix['WH','HR',c('WH', 'HH', 'HR',
 'WR', 'RR')] <- c(1-cF, 1+cF*chF, (cF*(1-chF)*crF + 1+cF*chF)+(cF*(1-chF)*(1-crF)),
 1-cF, (cF*(1-chF)*crF)+(cF*(1-chF)*(1-crF)))/4

 # female deposition things
 tMatrix['HH','WW', c('WH', 'HH', 'HR')] <- c( 1-dF, dF*dhF, (dF*(1-dhF)*drF)+(dF*(1-
dhF)*(1-drF)))

 tMatrix['HH','WR', c('WH', 'HH', 'HR')] <- c( 1-dF, dF*dhF, (dF*(1-dhF)*drF + 1)
+(dF*(1-dhF)*(1-drF)))/2

  tMatrix['HR','WW', c('WH', 'WR', 'HH', 'HR',
```

```
 'RR')] <- c( 1-dF, 1-dF, dF*dhF, (dF*(1-dhF)*drF)+(dF*(1-dhF)*(1-drF)),
(dF*drF)+(dF*(1-drF)))/2
 tMatrix['HR','WR', c('WH', 'WR', 'HH', 'HR',
 'RR')] <- c( 1-dF, 1-dF, dF*dhF, (dF*(1-dhF)*drF + 1)+(dF*(1-dhF)*(1-drF)), (dF*drF +
1)+(dF*(1-drF)))/4

 #male specific homing
 tMatrix['WW','WH', c('WW', 'WH', 'WR')] <- c(1-cM, 1+cM*chM, (cM*(1-
chM)*crM)+(cM*(1-chM)*(1-crM)))/2
 tMatrix['WR','WH', c('WW', 'WH', 'WR',
 'HR', 'RR')] <- c(1-cM, 1+cM*chM, (cM*(1-chM)*crM + 1-cM)+(cM*(1-chM)*(1-crM)),
 1+cM*chM, (cM*(1-chM)*crM)+(cM*(1-chM)*(1-crM)))/4
 tMatrix['HH','WH', c('WH', 'HH',
 'HR')] <- c((1-cM)*(1-dF), 1+cM*chM + (1-cM)*dF*dhF,
 (cM*(1-chM)*crM + (1-cM)*dF*(1-dhF)*drF)+(cM*(1-chM)*(1-crM) + (1-cM)*dF*(1-
dhF)*(1-drF)))/2

 tMatrix['RR','WH', c('WR', 'HR', 'RR')] <- c(1-cM, 1+cM*chM, (cM*(1-
chM)*crM)+(cM*(1-chM)*(1-crM)))/2

 tMatrix['HR','WH', c('WH', 'HH', 'HR', 'WR', 'RR')] <- c((1-cM)*(1-dF), 1+cM*chM + (1-
cM)*dF*dhF, (cM*(1-chM)*crM + 1+cM*chM + (1-cM)*dF*(1-dhF)*drF)+(cM*(1-
chM)*(1-crM) + (1-cM)*dF*(1-dhF)*(1-drF)), (1-cM)*(1-dF),(cM*(1-chM)*crM + (1-
cM)*dF*drF)+(cM*(1-chM)*(1-crM) + (1-cM)*dF*(1-drF)))/4

 #male stuff from female deposition
 tMatrix['WW','HH', 'WH'] <- 1
 tMatrix['WR','HH', c('WH', 'HR')] <- c( 1, 1)/2
 tMatrix['WW','HR', c('WH', 'WR')] <- c( 1, 1)/2
 tMatrix['WR','HR', c('WH', 'WR', 'HR', 'RR')] <- c( 1, 1, 1, 1)/4
```

Table S5. Fitness, GD, maternal deposition, and GDBI formation parameters used in the MGDrivE modeling for the AeaNosC109^{GD} and AeaZpgC109^{GD} lines. * = fitness cost defined for GD hemizygotes.

MGDrivE parameter	description	AeaNosC109^{GD}	AeaZpgC109^{GD}	Reference
**betaK**	Daily # of eggs laid per female mosquito	20.9	23.5	Sanchez *et al*., 2020 (3); Otero *et al*., 2006 (4)
**tEgg**	Number of days spent in the egg stage	5	5	Sanchez *et al*., 2020 (3); Christophers, 1960 (5)
**tLarva**	Number of days spent in the larval stage	9.5	9.5	This paper
**tPupa**	Number of days spent in the pupal stage	2	2	This paper
**popGrowth**	Population growth per generation	1.175	1.175	Sanchez *et al*., 2020 (3); Simoy *et al.,* 2015 (6)
**muAD**	Daily death rate for adult mosquitoes	0.09	0.09	Sanchez *et al*., 2020 (3); Fay, 1964 (7); Focks *et al*., 1993 (8); Horsfall, 1955 (9).
**cM**	Male homing rate	0.76	0.58	This paper
**chM**	Male correct homing rate	0.93	1	This paper
**crM**	Male gene drive resistance generating rate	0.07	0	This paper
**cF**	Female homing rate	0.81	0.69	This paper
**chF**	Female correct homing rate	0.93	0.97	This paper
**crF**	Female gene drive resistance generating rate	0.07	0.03	This paper
**dF**	Female deposition homing rate	0.19	0.14	This paper
**dhF**	Female deposition correct homing rate	0	0	This paper
**drF**	Female gene drive resistance generating rate as result of female deposition	1	1	This paper
**xiM**	Genotype-specific male pupatory success	HW* = 0.13	NULL	This paper
**xiF**	Genotype-specific female pupatory success	HW* = 0.13	NULL	This paper

**s**	Genotype-specific fractional reduction in fertility	0.1	0.1	This paper

Table S6. Polymorphisms in the active target sites for the Carb109 and TIMP-P4 loci among 132 genomes of *Aedes aegypti.* The polymorphisms are indicated in boldface, while the PAM sequences are underlined. Data taken from Schmidt *et al*., 2020 (10).

sgRNA	Polymorphisms (5' -protospacer + PAM -3')	Genomic locus*	Genome coverage	Polymorphism frequency
TIMP-P4	GACCAACGGCAGTCATTGTG T**T**G	2:321382218	230	0.026087
	GACCAAC**C**GCAGTCATTGTG TGG	2:321382231	222	0.00900901
Carb109	G**W**TATGCCGAAGAAAAGCCA GGG	3:409699154	264	0.0113636

**Aedes aegypti* genome, strain Liverpool, version Vectorbase-54: LVP_AGWG.

Table S7. Numbers of larval pools assessed and average metrics for GD inheritance among the OX-1 crosses.

Line	Cross	n groups	n total	Weighted average±SEM	Min	Max	n min	n max	Sample min	Sample max	Sample average (n) ±SEM
AeaNosT4^{GD}	Female	22	1131	46.6±5.3	40.3	56.3	67	64	22	106	51.4±4.8
	Male	24	2072	48.7±5.2	27.9	59.7	43	57	22	182	86.3±8.7
AeaNosC109^{GD}	Female	20	1380	73.3±12.3	48.7	94.0	37	67	23	142	69.0±6.1
	Male	20	1864	70.1±15.8	45.7	96.1	81	77	23	172	93.2±7.3
Aeaβ2tC109^{GD}	Female	10	409	48.2±4.0	43.4	55.0	53	20	20	63	40.9±4.6
	Male	15	1029	47.5±2.7	42.9	55.2	56	29	29	142	68.6±8.8
AeaZpgC109^{GD}	Female	13	711	66.1±9.8	48.3	80.4	58	51	34	77	54.7±4.0
	Male	19	1807	55.6±8.3	42.3	77.8	97	27	21	174	95.1±8.9

Table S8. Numbers of larval pools assessed and average metrics for GD inheritance among the OX-2 crosses.

Line	Cross	n groups	n total	Weighted average±SEM	Min	Max	n min	n max	Sample min	Sample max	Sample (n) average±SEM
AeaNosT4^{GD}	FFH	26	1451	49.4±18.9	33.3	89.5	21	124	21	124	55.8±4.9
	FFL	29	1441	47.7±6.4	38.1	60.0	21	25	21	113	49.7±4.6
	FMH	23	795	48.4±8.9	40.0	72.0	25	25	21	58	34.6±2.5
	FML	24	1219	47.4±7.4	34.8	54.2	23	59	23	110	50.8±3.8
	MFH	31	1894	46.5±9.0	26.6	55.3	79	47	25	98	61.1±4.1
	MFL	30	2350	48.1±6.6	39.2	68.4	51	57	24	156	78.3±5.2
	MMH	27	1735	48.8±5.3	38.6	60.0	44	20	20	120	64.3±5.8
	MML	28	2521	49.2±4.7	37.9	58.6	66	29	22	195	90.0±10.3
AeaNosC109^{GD}	FFH	28	1971	68.4±29.0	43.0	91.4	107	140	20	151	70.4±7.3
	FFL	29	2032	66.6±32.4	43.5	100.0	69	114	29	114	70.1±4.7
	FMH	22	1775	69.7±28.1	46.1	95.9	89	49	23	200	80.7±8.0
	FML	27	1690	57.4±24.0	22.9	93.9	35	65	22	135	62.6±6.9
	MFH	27	2287	65.9±29.8	40.0	100.0	105	44	26	223	84.7±11.2
	MFL	26	2636	76.4±37.0	42.9	100.0	77	137	24	237	101.4±12.3
	MMH	28	2734	72.7±35.4	36.8	100.0	76	20	20	232	97.6±9.8
	MML	32	3162	62.4±25.7	36.0	98.1	75	53	20	212	98.8±9.6
AeaZpgC109^{GD}	FFH	16	1129	56.9±9.9	40.0	78.8	40	33	20	167	70.6±10.4
	FFL	21	1413	58.5±21.6	30.4	89.2	23	37	22	164	67.3±10.3
	FMH	22	1651	58.7±13.5	42.1	79.3	38	87	27	176	75.1±8.5
	FML	16	1017	58.4±17.2	34.8	79.7	23	59	23	137	63.6±8.2
	MFH	20	1348	56.7±19.4	39.5	84.7	86	111	21	174	67.4±9.2
	MFL	20	1531	59.4±19.5	39.8	90.9	103	22	21	179	76.6±10.4
	MMH	23	1925	54.2±21.7	27.7	91.9	137	74	20	176	83.7±11.3
	MML	19	1308	52.2±25.7	19.1	81.9	21	177	21	177	68.8±10.9

Table S9. Percentage of amplicons containing gene drive blocking indels (GDBI) for pooled negative larvae from the OX-2 generations.

Line	Cross	n groups	Median (%)	Q1 (%)	Q3 (%)	Min (%)	Max (%)
AeaNosT4^{GD}	FFH	10	0.020	0.017	0.025	0.01	0.07
AeaNosT4 ^{GD}	FFL	11	0.021	0.019	0.026	0.01	4.2
AeaNosT4 ^{GD}	FMH	11	0.117	0.051	0.54	0.02	1.5
AeaNosT4 ^{GD}	FML	10	0.040	0.025	0.158	0.01	2.8
AeaNosT4 ^{GD}	MFH	10	0.018	0.016	0.022	0.01	0.03
AeaNosT4 ^{GD}	MFL	10	0.021	0.019	0.024	0.01	0.03
AeaNosT4 ^{GD}	MMH	11	0.019	0.015	0.024	0.01	0.03
AeaNosT4 ^{GD}	MML	12	0.022	0.017	0.026	0.01	0.55
AeaNosC109 ^{GD}	FFH	10	19.1	4.9	32.33	0.71	98.1
AeaNosC109 ^{GD}	FFL	9	12.4	11.5	22.24	6.0	53.9
AeaNosC109 ^{GD}	FMH	5	4.7	2.4	10.26	0.79	25.9
AeaNosC109 ^{GD}	FML	9	11.9	8.0	18.15	0.06	26.5
AeaNosC109 ^{GD}	MFH	9	0.15	0.06	1.70	0.02	14.8
AeaNosC109 ^{GD}	MFL	10	9.2	2.8	25.23	0.19	68.0
AeaNosC109 ^{GD}	MMH	9	1.6	0.16	63.94	0.03	92.0
AeaNosC109 ^{GD}	MML	10	14.1	0.36	38.39	0.02	100.0
AeaZpgC109 ^{GD}	FFH	8	2.1	1.1	3.48	0.02	4.5
AeaZpgC109 ^{GD}	FFL	11	2.6	1.5	17.87	0.09	53.9
AeaZpgC109 ^{GD}	FMH	11	2.0	0.56	12.15	0.03	21.5
AeaZpgC109 ^{GD}	FML	5	11.9	2.9	13.37	0.03	14.2
AeaZpgC109 ^{GD}	MFH	9	0.04	0.03	0.19	0.02	3.8
AeaZpgC109 ^{GD}	MFL	8	1.6	0.04	2.04	0.01	22.7
AeaZpgC109 ^{GD}	MMH	11	0.02	0.01	1.46	0.01	6.7
AeaZpgC109 ^{GD}	MML	9	0.07	0.01	0.63	0.00	14.6
HWE	Carb109	1	0.04%				
HWE	TIMP-P4	1	0.02%				

SI References

1. Dang Y, Jia G, Choi J, Ma H, Anaya E, Ye C, Shankar P, Wu H. 2015. Optimizing sgRNA structure to improve CRISPR-Cas9 knockout efficiency. Genome Biol. 16:280.
2. Basu S, Aryan S, Overcash JM, Samuel GH, Anderson MAE, Dahlem TJ, Myles KM, Adelman ZN. 2015. Silencing of end-joining repair for efficient site-specific gene insertion after TALEN/CRISPR mutagenesis in Aedes aegypti. Proc Natl Acad Sci USA. 112(13):4038-4043.
3. Sánchez C HM, Wu SL, Bennett, JB, Marshall, JM. 2020. MGDRIVE: A modular simulation framework for the spread of gene drives through spatially explicit mosquito populations. Methods Ecol Evol. 11:229–239.3.
4. Otero M, Solari HG, Schweigmann N.2006. A stochastic population dynamics model for *Aedes aegypti*: formulation and application to a city with temperate climate. Bull Math Biol. 68(8):1945-1974.
5. Christophers SR. 1960. *Aedes aegypti:* the yellow fever mosquito. 1st ed., Cambridge University Press, London, UK.
6. Simoy MI, Simoy MV, Canziani GA. 2015. The effect of temperature on the population dynamics of *Aedes aegypti*. Ecological modeling 314:100-110.6.
7. Fay, R. W. 1964. The biology and bionomics of *Aedes aegypti* in the laboratory. *Mosquito News* 24(3):300-308.
8. Focks DA, Haile DG, Daniels E, Mount GA. 1993. Dynamic life table model for *Aedes aegypti* (Diptera: Culicidae): Analysis of the literature and model development. J Med Entomol. 30(6):1003-1017.
9. Horsfall WE. 1955. Mosquitoes - their bionomics and relation to disease. The Ronald Press Co., New York, NY.
10. Schmidt H, Collier TC, Hanemaaijer MJ, Houston PD, Lee Y, Lanzaro GC. 2020. Abundance of conserved CRISPR-Cas9 target sites within the highly polymorphic genomes of *Anopheles* and *Aedes* mosquitoes. Nat Commun. 11:1425.
